# An Overview of The Globozoospermia as A Multigenic Identified Syndrome 

**DOI:** 10.22074/ijfs.2019.5561

**Published:** 2018-10-02

**Authors:** Parastoo Modarres, Marziyeh Tavalaee, Kamran Ghaedi, Mohammad Hossein Nasr-Esfahani

**Affiliations:** 1 Department of Reproductive Biotechnology, Reproductive Biomedicine Research Center, Royan Institute for Biotechnology, ACECR, Isfahan, Iran; 2 Department of Biology, Faculty of Sciences, University of Isfahan, Isfahan, Iran; 3 Department of Cellular Biotechnology, Cell Science Research Center, Royan Institute for Biotechnology, ACECR, Isfahan, Iran; 4 Isfahan Fertility and Infertility Center, Isfahan, Iran

**Keywords:** Acrosome, Globozoospermia, Male Infertility

## Abstract

Acrosome plays an integral role during fertilization and its absence in individuals with globozoospermia leads to failure of *in vitro* fertilization (IVF) and oocyte activation post-intracytoplasmic sperm injection (ICSI). A variety of processes, organelles and structures are involved in acrosome biogenesis including, trans-golgi network (TGN), acroplaxome and cellular trafficking. This review aims to explain roles of related signals and molecules involved in this process and also describe how their absence in form of mutation, deletion and knockout model may lead to phe- nomenon referred to globozoospermia.

## Introduction

Fertilization is a multifactorial process for fusion of gametes to initiate development of a new individual. For successful fertilization to occur, each process needs to take place in a coordinated manner. One of the main steps of fertilization is acrosome reaction during which proteolytic contents of acrosome is released to facilitate zona binding and penetration to zona and the oocyte by sperm (1). Structural and functional anomalies of acrosome lead to inability of sperm to penetrate oocyte, thereby resulting in failed fertilization and infertility. Furthermore, different studies show that when barriers of fertilization are bypassed during intra-cytoplasmic insemination, in certain cases with acrosome anomalies, the ability of sperm to induce fertilization is still diminished due to inability of the oocyte to induce activation (2-4).

Total absence of acrosome was first reported by Schirren et al. (5) which manifested by round head spermatozoa appearance. This syndrome has termed “globozoospermia” with a prevalence of 0.1% among infertile male population and two subtypes: complete (type-I: 100% spermatozoa are round head) or partial (type-II: over 50% spermatozoa are round head). Further genetic pedigree analysis revealed genetic basis with possible autosomal recessive inheritance is responsible for incidence of this syndrome (6). Individuals with globozoospermia commonly show no mental and physical abnormities, and generally have normal karyotype with no micro-deletion in chromosome Y (7). However, sperm cells of the affected persons are acrosome-less, and incapable of penetrating in zona pellucida (ZP). Considering the importance of acrosome in fertilization and oocyte activation, this review aimed to describe the genetic and molecular aspects of globozoospermia.

### Genetic aspects of globozoospermia

Literature survey shows different approaches were taken by various researchers to detect genetic and molecular basses of globozoospermia. These approaches include: i. Purposefully designed knockout mice for a variety of genes including: *Hrb, Zpbp1, Hsp90b1, Vps54, SAMP32 (SPACA1)*, ii. Knockout mice approach for different purpose which later exhibited globozoospermia manifestation. The target genes were as: *Csnk2a2, GOPC, Gba2, PICK1*, iii. Whole-genome scan analysis which were carried out using SNP-array approach on the genome of globozoospermia and the genes associated with this syndrome. These genes identifiers are: *SPATA16* and *DPY19L2,* and iv. Assessment of protein localization associate with acrosome biogenesis such as: SPGL4, Calicin.

Among the 13 genes involved in globozoospermia, they were mostly related to Golgi network, acrosome formation, sperm head shaping (anchorage of acrosome to nucleus) and zona binding. Only, four genes have been so far detected in individuals presenting globozoospermia including *DPY19L2, SPATA16, PICK1* and *Calicin* (6-10). It is important to note that in addition to genetic defects, deregulation of proteins (up or down regulation) can also result in the onset of globozoospermia. To further elucidate the role of these 13 genes, below section provides the cellular and molecular mechanisms in acrosome biogenesis. 

### Acrosome biogenesis

**Fig.1:**
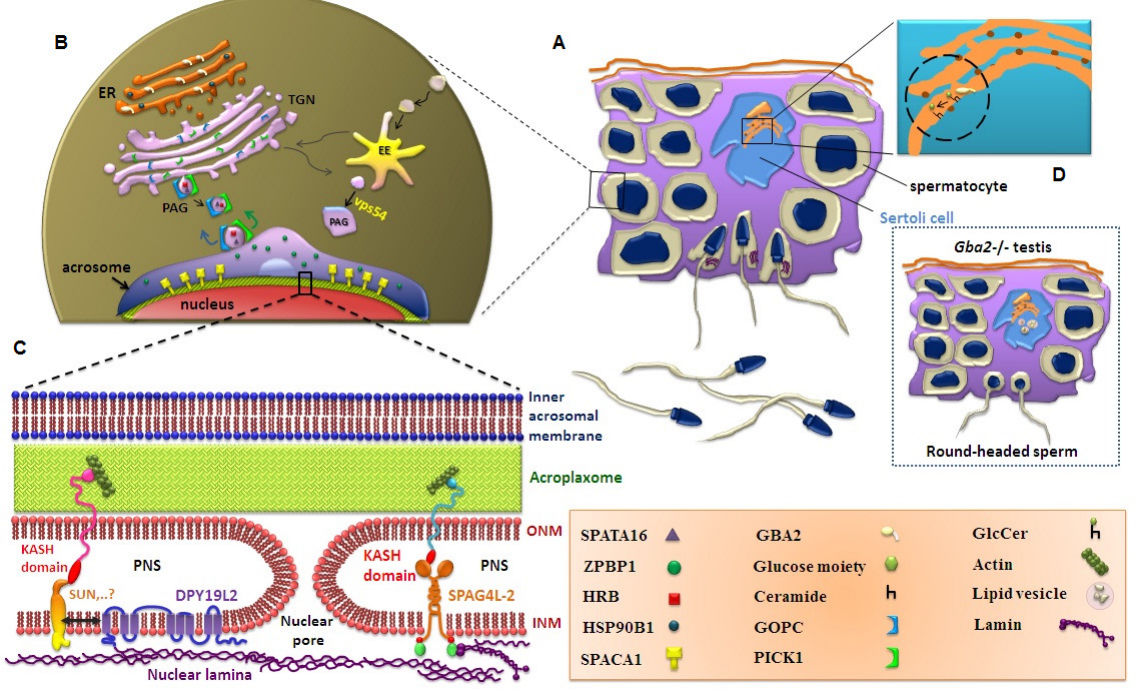
Schematic illustration of subcellular distribution and localization of several proteins involved in acrosome biogenesis in germ cells during spermiogenesis. A. Lack/disruption of each protein leads to impaired acrosome formation, and characterized as globozoospermia, B. SPATA16 and HRB are proteins which are transported in proacrosomal vesicle from Golgi to acrosome via PAG transporter proteins like GOPC and PICK1. Vps54 has a possible role in tethering of vesicles from early endosomes (EE) to the trans-Golgi network (TGN). SPACA1 is a transmembrane protein localized in inner acrosomal membrane of developing spermatozoa. Spaca1 deficiency leads to failure of acrosome thinning coincides with instability/or loss of acroplaxome and nuclear plate, C. DPY19L2 and SPAG4L/4L-2 are transmembrane proteins located on inner nuclear membrane (INM) and are likely to be participating to form linkage to nucleo- and cytoskeleton complex (LINC) complex. PNS: perinuclear space, and D. GBA2 is a non-lysosomal glycosylceramidase (GlcCer) involved in catalysis of glycolipids, leading to releasing glucose moiety in ER lumen. Right inset: accumulation of lipid vesicles in testicular Sertoli cell of Gba2-null mice.

Acrosome structure is divided into two segments, anterior and equatorial segments. The former segment contains enzymes that are released during acrosome reaction while the latter segment is predominantly involved in sperm-oocyte fusion. Biogenesis of acrosome begins during meiosis and continues through early stages of spermiogenesis which is divided into four steps including golgi, cap, acrosomal and maturation phases (Fig .1A) (11). In golgi phase, pro-acrosomal granules (PAGs) derived from endoplasmic reticulum (ER) are transported to golgi sacs through anterograde pathway. Subsequently, PAGs are transported toward sperm nucleus where they bind to an actin-keratin containing cytoskeletal plate termed “acroplaxome”. In cap phase, PAGs fuse with each other to form a structure known as “acrosomal cap”. In acrosomal phase, cap begins to spread over anterior part of nucleus to form an acrosome like structure. In maturation phase, following condensation and elongation of nucleus with the help of manchette, the equatorial segment of acrosome is shaped. At this stage, the acrosome is surrounded by two distinct membranes known as “inner” and “outer” acrosomal membranes. Inner acrosomal membrane locates in vicinity of nuclear membrane, tightly anchors the acrosome to the nuclear envelop through cytoskeletal components known as “perinuclear theca” (Fig .1B, C) (12).

Originally, acrosome was described as a modified lysosome while recent literatures suggest that in addition to PAGs forming from Golgi network, early endosome (EE) may also have a role in acrosomal biogenesis (Fig .1B). Hence it is agreed that cargos originated from Golgi apparatus are sorted to plasma membrane, subsequently are recruited back into cytoplasm and incorporate into developing pro-acrosomes (13). During acrosomal biogenesis, particular proteins are involved that their absence or defect may result in globozoospermia.

### Globozoospermia and associated proteins

#### Csnk2a2

Casein kinase IIα' polypeptide (Csnk2a2) was the first introduced protein whose gene was associated with globozoospermia. This protein is a kind of serine-threonine kinase which relates to nuclear matrix. Multiple forms of acrosome imperfection like complete lack of acrosome, indented/detached acrosome from nucleus, and acrosomal remnants were recognized in spermatozoa of *Csnk2a2*-deficient mice. In other words, mice lacking the *Csnk2a2* gene demonstrated aberrant development in both nucleus and acrosome (14).

#### GBA2

β-Glucosidase 2 (GBA2) is a glycolipid hydrolase resident in ER and its relation to globozoospermia was first recognized in glycolipid storage disease due to deficiency of *Gba2* in male mice with reduced fecundity. Glucosylceramides are normally transferred from developing germ cells to Sertoli cells for subsequent breakdown. Loss of the GBA2 results in accumulation of glucosylceramide in Sertoli cells and disrupts transport of glycolipid from germ cells which in turn interrupts normal Sertoli-germ cell interactions. Therefore, this defect leads to formation of abnormal sperm (Fig .1A, D). Unlike in mice, mutational assessments for *GBA2* in 3 unrelated families, originating from Britain, Canada, and Germany, have been unfruitful to show an association with globozoospermia in man (15).

#### SPATA16

Spermatogenesis-associated 16 (SPATA16), also known as NYD-SP12, is a human testis specific protein and its ortholog encoding gene is expressed in mouse spermatocyte and spermatids. SPATA16 has a subcellular localization in Golgi apparatus and pro-acrosmal vesicles being transported to acrosome. Its function is sorting and modification of acrosomal enzymes in Golgi network (16). This protein also interacts with other proteins involved in acrosomal biogenesis including GOPC and Hrb (Fig .1B). *SPATA16* was the first gene which was shown to contribute to human globozoospermia with an autosomal dominant pattern of inheritance (6).

#### Hrb

Hrb, formerly known as human Rev-binding/interacting protein (hRIP), is the cofactor of HIV-1 Rev protein, involved in shuttling of proteins between nucleus and cytoplasm. Hrb interacts with proteins involved in nucleo-cytoplasmic trafficking (17). Based on these functions, *Hrb* mice knockout model revealed that, Hrb is involved in vesicle to vesicle docking, fusion of pro-acrosmal vesicles with acrosome and thereby acrosomal biogenesis (Fig .1B). Therefore, its absence was associated with globozoospermia (18). Further analysis of *Hrb-/-* mice revealed a second role for Hrb in formation of acroplaxome plague. Acroplaxome is encompassed by 3 proteins including: F-actin, Sak57 (an ortholog of keratin5) and myosin Va. In *Hrb* deficient mice, keratin 5 filament bundle in acroplaxome is missing and the strength of acrosome vesicle in binding to nucleus is reduced which its outcome is manifested as globozoospermia (19).

#### PICK1

Protein interacting with C kinase 1 (PICK1) was initially found in brain. It plays an important role in protein trafficking of neurons. Although the PICK1-/- mice were produced to study the brain function but these mice were infertile. PICK1 like GOPC has a postsynaptic density 95, discs large, and zonula occludens-1 (PDZ) domain which is involved in PAG trafficking (Fig .1B) (20). So far one mutation in this gene has been reported to be associated with globozoospermia (9).

#### GOPC

*GOPC* gene, encodes Golgi-associated PDZ and coiled-coil motif containing protein (GOPC). GOPC protein has 5 domains including: one PDZ domain, two coiled-coil motifs, and two conserved domains with unknown function (21). GOPC is involved in PAG transport from Golgi network to acrosome and its absence (GOPC-/) is associated with globozoospermia (Fig .1B). In addition to lack of acrosome, other deformities associated with this defect, are lack of post-acrosomal sheath or peri-nuclear theca (22) and coiled-coil tail (23).

#### ZPBP1

ZP binding protein 1 (ZPBP1) or Sp38 or Iam 38 and its paralog, ZPBP2, were described as acrosomal proteins in mice and human. *ZPBP1-* deficient male mice are sterile and present round-head spermatozoa due to disrupted acrosome biogenesis. Zpbp1 is an intra-acrosomal protein and Zpbp1-deficient spermatids demonstrate defective protein matrix assembly and results in fragmentation of the abnormal acrosomes (Fig .1B) (24).

Considering candidate genes responsible for abnormal sperm head morphology, heterozygous mutation in ZPBP1 were described in patients with aforementioned condition, however direct involvement of ZPBP1 in the onset of such conditions remains to be clarified (25).

#### SPACA1

Sperm acrosome associated 1 (SPACA1) or SAMP32 (sperm acrosomal membrane-associated protein 32) is a testis-specific transmembrane protein involved in sperm-egg interaction. During elongation stage of developing spermatozoa, this protein is localized in inner acrosomal membrane (Fig .1B) (26) and no role has been envisaged in acrosome reaction. The role of this protein in globozoospermia was initially recognized when this protein was absent in Gopc- and Zpbp1-disrupted mouse line. However, later studies revealed that “disruption of Gopc caused a significant decrease in SPACA1 and ZPBP1” while “disruption of *Zpbp1* caused loss of SPACA1whereas GOPC was unaffected” and “disruption of Spaca1 did not affect the amounts of GOPC and ZPBP1 in the testis”. Thereby, suggesting that Spaca1 is likely downstream of these two genes. *Spaca1* deficiency leads to failure of acrosome thinning, coinciding with instability/or loss of acroplaxome and nuclear plate (27) and unlike most of aforementioned proteins, it has no role in protein transport in golgi network or in acrosome formation.

#### Hsp90b1

Heat shock protein 90b1 (Hsp90b1), a member of heat shock protein 90 family, is a testis specific endoplasmic chaperone involving in entire folding, activation and/or degradation of ER proteins (Fig .1B). It was shown that *Hsp90b1*- null sperm cells are round and not able to fertilize the oocyte. Therefore, absence of this protein showed a potential role in the incidence of globozoospermia (28). Recent study has hypothesized that phosphorylation of Hsp90b1 along with other chaperon proteins during sperm capacitation leads to the formation of ZP -recognized protein complexes and/or the translocation of these complexes to the surface of spermatozoa (29).

#### Vps54

Vps54 is a protein apparently involved in tethering of vesicles from endosomes to the trans-golgi sacs (13). This is an alternative pathway in acrosome biogenesis as mentioned earlier. The role of this protein in acrosomal biogenesis was found when wobbler mouse with *Vps54(L967Q)* mutation were found to cause sterility. The protein codified by the Vps54 gene, has an active role in vesicular retrograde trafficking and like Hrb gene affects proacrosomal vesicle coalesces with acrosome (Fig .1B) (30).

#### SPAG4L/4L-2

SPAG4L and its isoform, are testis specific proteins, belong to SUN domain proteins. These transmembrane proteins are located on inner nuclear membrane (INM). By interacting with their partner on outer nuclear membrane (ONM), known as KASH domain can anchor or create linkage to nucleo- and cytoskeleton complex (LINC complex) (Fig .1C) (31).

Different members of this anchoring system have been discovered but their role in acrosomal biogenesis remains to be determined. Among these proteins, absence of SPAGL4/4L-2 has been associated with globozoospermia. SPAG4L/4L-2 is localized on apical side of nuclear membrane of developing spermatid and it may have a function in docking of acrosome vesicle to nuclear membrane (31).

#### DPY19L2

DPY19L2, similar to SPAGL4/4L-2, is a transmembrane protein with 8-10 predicted domains in inner nuclear membrane. The expression of this protein is restricted to testis and like SPAGL4 (or SUN5) is involved in anchorage of cytoskeleton to nuclear membrane (Fig .1C). Therefore, its absence leads to instability and dissociation of the layered structure of acroplaxome, the nuclear/acrosome bridging region. Thereby, its absence results in formation of round head spermatozoa (32). ElInati et al. (10) revealed that *DPY19L2* gene has an inevitable relationship with globozoospermia. They have shown that *DPY19L2* is one of the main genes responsible for globozoospermia. In this regard, a wide spectrum of plausible mutations for this gene has been detected in globozoospermic individuals such as: deletion of the whole locus, nonsense, missense, splicing mutations, partial deletion in different regions of the gene encompassing exons 8, 9, 11, 15, 21 and intron 11 (10, 33-36).

#### Calicin

Calicin is one of the subacrosomal cytoskeletal proteins involved in acrosomal biogenesis which its absence results in globozoospermia (8).

### The proteomics of round-head spermatozoa

Collectively, it is evident that numerous proteins are involved in acrosomal biogenesis and the absence of each protein may result in globozoospermia phenotype. One approach to distinguish proteins associated with globozoospermia is comparative proteomics between normozoospermia and globozoospermia. The results of this study have shown up/down regulation of several proteins in affected subjects. Spermatozoa acrosome membrane-associated protein 1 (SAMP1) and sperm protein associated with the nucleus on the X chromosome (SPANX) are among the proteins that their expression was shown to be down regulated (37). SAMP1 is a glycoprotein receptor residing in inner nuclear membrane and its absence results in mislocalization of the SUN1 (38). SPANX also acts as a nuclear envelope protein residing in post-acrosomal perinuclear theca and is expected to be associated with acrosome-nucleus binding and down regulation of this protein in globozoospermia may be underlying cause of the lack of acrosome (37).

## Conclusion

Taken together, the results of this study suggest that mutation, deletion of genes products associate with Golgi apparatus, formation of acroplaxome or those associated with neuclo-cytoskeleton involved in attachment of acrosome with nucleus have a potential role in induction of globozoospermia.
